# Oral Health and Quality of Life in People with Autism Spectrum Disorder

**DOI:** 10.3390/jcm13175179

**Published:** 2024-08-31

**Authors:** Antonio Fallea, Luigi Vetri, Simona L’Episcopo, Massimiliano Bartolone, Marinella Zingale, Eleonora Di Fatta, Gabriella d’Albenzio, Serafino Buono, Michele Roccella, Maurizio Elia, Carola Costanza

**Affiliations:** 1 Oasi Research Institute-IRCCS, Via Conte Ruggero 73, 94018 Troina, Italy; afallea@oasi.en.it (A.F.); lvetri@oasi.en.it (L.V.); slepiscopo@oasi.en.it (S.L.); mbartolone@oasi.en.it (M.B.); mzingale@oasi.en.it (M.Z.); edifatta@oasi.en.it (E.D.F.); fbuono@oasi.en.it (S.B.); melia@oasi.en.it (M.E.); 2 School of Computing, Queen’s University, Kingston, ON K7L 3N6, Canada; 3 The Intervention Centre, Oslo University Hospital, 0313 Oslo, Norway; 4 Department of Psychology, Educational Science and Human Movement, University of Palermo, 90133 Palermo, Italy; michele.roccella@unipa.it; 5 Department of Sciences for Health Promotion and Mother and Child Care “G. D’Alessandro”, University of Palermo, 90133 Palermo, Italy; carola.costanza@unipa.it

**Keywords:** oral health, quality of life, autism spectrum disorder (ASD), oral hygiene

## Abstract

This article delves into the intricate relationship between oral health, quality of life, and behavioral characteristics in individuals with autism spectrum disorder (ASD). **Background/Objectives:** Autism has been associated with various challenges, and this study seeks to elucidate the impact of ASD on oral health outcomes and overall well-being. The research focuses on assessing overall oral health by evaluating various parameters, such as the condition of lips, tongue, gums and tissues, natural teeth, dentures, oral hygiene, and dental pain in individuals with ASD. Additionally, the study explores the influence of age, sex, and certain variables, like basic daily living skills on oral health practices, aiming to identify potential correlations between these factors and oral health outcomes. **Methods:** We employed standardized instruments to quantitatively measure and analyze the impact of oral health status on the overall quality of life experienced by individuals with ASD. **Results:** The study found a statistically significant positive association between oral health, measured by the Oral Health Assessment Tool (OHAT), and quality of life, as indicated by EuroQol 5-Dimensions Youth version (EQ-5D-Y) total scores (β = 0.13045, *p* = 0.00271). This suggests that better oral health is linked to higher quality of life. When adjusting for age and sex in a multiple linear regression model, the association remained significant but with a slightly reduced effect size (β = 0.10536, *p* = 0.0167). Age also showed a marginally significant positive association with quality-of-life scores. ANOVA results indicated that participants with advanced oral health status reported significantly higher quality-of-life scores than those with poorer oral health (*p* = 0.00246). The study also found that intelligence quotient (IQ) does not substantially influence dental health status, while the “Autonomy” subscale of the EQ-5D-Y is positively related to the OHAT. **Conclusions:** Unhealthy oral conditions significantly impact the overall quality of life in individuals with ASD. Therefore, it is crucial to include regular dental assessments and treatments in therapeutic protocols for patients with ASD.

## 1. Introduction

It is estimated that about 1 in 100 children worldwide are affected by autism spectrum disorder (ASD) [1]. ASD is a neurodevelopmental disorder (NDD), defined as a range of conditions characterized by social, emotional and communication problems alongside repetitive behaviors, difficulty in changing daily activities, and multimodal sensory abnormalities [2]. NDDs, in general, have a negative impact on oral health-related quality of life [3], and oral health problems are more common in children with NDDs than in typically developing ones [4]. Children with neurodevelopmental disorders are at greater risk of developing insufficient oral hygiene due to difficulties in taking care of themselves, the presence of behavioral abnormalities and, sometimes, sensory atypia [3,4,5]. People with ASD have varying degrees of alteration in sensory and stimulus processing [6,7,8]. This typical feature also complicates the educational aspect of a correct oral hygiene procedure due to the difficulty that many patients present in relation to physical contact, even from their parents [8]. Some typical traits of ASD affect the ability to carry out activities of daily living independently, such as self-care and personal and oral hygiene. Many children depend on their caregivers to perform these activities [9].

Moreover, children with ASD also often require long-term medications or supplements, which are more easily administered in the form of syrups. The use of oral formulations, usually sweetened, increases the risk of tooth decay [10,11,12]. Recently, several systematic reviews and meta-analyses have demonstrated a higher burden of dental plaque and an increased risk of dental caries in children and adolescents with ASD [13,14,15]. The low tolerance for frustration and the difficulty of adapting to new settings make trying to consult a dental specialist at an early age difficult [16]. Children with ASD who show sensitivity to noisy stimuli or discomfort with loud noises may also have varying degrees of intolerance to sights and sounds in the dental setting [17,18]. The reactions can be exaggerated and unusual, explode into fits of agitation, and make the operations dangerous enough that general anesthesia, even for simple dental hygiene operations, is sometimes required as a management option [4,19].

For example, it is important to note that there are specific techniques for approaching the oral health of this type of population. Interventions aimed at facilitating dental care in ASD patients, such as a sensory-adapted dental environment (SADE), lighting effects, and vibroacoustic stimuli, have been suggested [20]. It has been shown that these environments can not only facilitate surgery but also reduce anxiety and induce a state of relaxation [21,22,23]. Behavioral interventions, such as gradual exposure to fearful stimuli, distraction/relaxation, positive reinforcement, and desensitization strategies (especially for children with ASD with sensory problems), allow them to be able to approach these problems [9].

It is well documented that both tooth decay and periodontal disease, which are the leading causes of impaired oral health, can worsen quality of life. A study on 1200 adults in Sweden showed that treatment costs related to dental caries have a significant impact on people’s quality of life (HRQoL). Although only 21.7% agreed to take part in the study (79 patients with active caries and 179 patients with inactive caries aged between 20 and 65 years), through the EQ-5D-5 L tool, there were problems relating to the anxiety/depression dimension, especially in the group with active caries. These results highlight the need for prevention strategies, as well as adequate allocation of funds in dentistry [24]. Furthermore, a study carried out on 350 preschool children (from 3 to 6 years; mean age 4.73) and their parents demonstrated a significant relationship between the score of a dmft index (decayed, missing and filled teeth) and the Early Childhood Oral Health Impact Scale (ECOHIS), showing how childhood dental caries can impact the oral health-related quality of life of children and their parents [25]. In particular, there are results from previous studies that investigated health-related quality of life through the HrQoL scale and oral health-related quality of life through the OHrQoL scale, especially our study in ASD patients. The sample consisted of 510 preschool children (253 with ASD and 257 controls). Compared to our study, as is known, a significant difference was also detected in the PedsQL (*p* < 0.001) and Early Childhood Oral Health Impact Scale (ECOHIS) scores (*p* < 0.001) in children with and without ASD. In regression analyses, the presence of ASD was associated with a greater probability of having PedsQL scores, a test that evaluates the quality of life related to health and oral health; lower (OR 0.10, 95% CI 0.06–0.15, *p* < 0.001) and higher ECOHIS scores (OR 2.34, 95% CI 1.60–3.42, *p* < 0.001). These results appear in line with our study but need to be further explored in order to identify relevant impacting aspects.

In a recent systematic review aimed at exploring parents’ perceptions of oral health related to quality of life in children and adolescents with autism, it emerged that, in a total of 15 included studies, parents’ perceptions of OHRQoL were poor and the most significant perceived impact concerned symptoms such as bad breath, food deposits, mouth breathing and nocturnal grinding, which undermine the child’s functional and social well-being aspects [26].

### Aims of the Study

The present study aimed to evaluate whether oral health influences the quality of life of a sample of children and adolescents with ASD, defined according to the Diagnostic and Statistical Manual of Mental Disorders, Fifth Edition (DSM-5) criteria (Table 1) [2]. We have hypothesized that people with autism spectrum disorder who have insufficient oral health have negative repercussions on their quality of life and have a higher frequency of behavioral abnormalities. The results of the present study will allow the implementation of measures to contrast poor oral health status in children with autism, such as improving caregivers’ awareness of children with ASD and increasing periodic routine dental examinations.

## 2. Materials and Methods 

### 2.1. Participants

A total of 163 children and adolescents with ASD, aged between 2 and 35 (129 males and 34 females; mean chronological age, 7.8 years) were included in the study. Our group consisted of toddlers (1–3 years old) and preschoolers (3–5 years old) = 71 subjects; school-age children (5–12 years old) and adolescents (12–17 years old) = 80 subjects; and young adults (18–36 years old) = 12 subjects. They were enrolled between March 2023 and January 2024 by selecting patients referred to Oasi Research Institute–IRCCS (Troina, Italy) and to the Department of Psychological, Pedagogical, Physical Exercise and Training Sciences of the University of Palermo. All participants or their legal guardians were informed about the study through verbal and written means and provided written informed consent before participating in the examination.

A multidisciplinary team diagnosed all participants, following the DSM-5 criteria. A comprehensive study focusing on the quality of life and oral hygiene in patients with ASD requires the involvement of a multidisciplinary team. This team ensures that various aspects of the patient’s health and well-being are addressed holistically. The key professionals involved and their roles are as follows: (a) one dentist who performed the clinical dental examination and assessed the OHAT (dentists are essential for identifying and managing oral health problems, which are common in patients with ASD due to challenges in maintaining oral hygiene); (b) two dental assistants who supported managing young patients and ensured that the sensory-adapted dental room was appropriately organized and utilized; (c) two psychologists who played a crucial role in the study by administering standardized tests to support and confirm the diagnosis of ASD (i.e., Childhood Autism Spectrum Disorder Test (CASD) and the Autism Diagnostic Observation Schedule second edition (ADOS-2)) [27]. These tools are widely recognized for their reliability and validity in diagnosing ASD. They help in systematically assessing behaviors and symptoms associated with autism; (d) two child neuropsychiatrists for the evaluation and treatment of neuropsychiatric conditions in children with ASD, providing insights into neurological and psychiatric aspects that may impact oral hygiene and overall health. 

As already stated, diagnoses were further confirmed using two of the most common diagnostic scales, namely ADOS-2 and CASD. Some of these patients had comorbid intellectual disabilities (ID) of varying degrees [28]. Due to the severity of the behavioral characteristics or non-compliance, IQ was not possible to estimate for all subjects. For 90 subjects, IQ level was calculated using the following tests: Wechsler [29] scale, Leiter 3 [30], and CTONI-2 [31]. For patients up to 3 years of age, we evaluated the developmental quotient using Griffith III [32]. This group’s IQ ranged from 44 to 141 (median IQ = 72.87). 

### 2.2. Procedure

We primarily used ADOS-2 and CASD to standardize measurements and categorize patients by the severity of autistic symptoms (Checklist for Autism Spectrum Disorder).

ADOS-2 is a tool for the behavioral assessment of ASD that allows a semi-structured and standardized evaluation of communication, social interaction, play, and restricted and repetitive behaviors through a series of activities that directly elicit behaviors related to the diagnosis. ADOS-2 includes five different modules, each of which takes 40 to 60 min to administer. The subject to be evaluated will be offered only one module, selected on the basis of chronological age and level of expressive language. Each module involves the examiner in a series of activities that require the use of the interactive stimulus materials in the kit. For the ADOS test, the cut-off values are reported on the protocols and are different based on the module used and the presence/absence of adequate expressive language. The average score of our patients was 20.4, which exceeded the cut-offs foreseen by the different modules. For module 1, the cut-off is 16 for subjects with “few to no words” and 12 for subjects with “some words”. For module 2, the cut-off is 9 for children aged 5 and over and 10 for children under age 5. For module 3, the cut-off is 9. For module 4, the cut-off is 10. For the toddler module test, there are risk bands that concern the probability that there may be an autism spectrum disorder (from 0 to 9, no risk for all small and older children with few or no words; from 0 to 7, no risk for children bigger with some words).

CASD is a screening tool that can also be adopted in the diagnostic process for evaluating ASD in children and adolescents aged between 1 and 16. CASD evaluates 30 symptoms of autism, covering six domains: problems with social interaction, perseverance, somatosensory disorders, atypical communication and development, humor, and attention and safety issues. Symptoms are rated as present or absent based on information from caregivers. It was impossible to administer CASD to all patients because some of them (13) were more than 16 years old. Furthermore, some of their parents or caregivers needed help to provide reliable information. In the case of the CASD test, the values are 7 or lower = range of scores within normal range; 8–10 = range of scores corresponding to a risk condition; 11–14 = borderline score range; 15–30 = range of scores corresponding to autism. The average score of our patients was 18.77 (8 patients had a score between 9 and 14; 95 patients had a score of 15 to 27).

The quality-of-life levels of the child with ASD were determined using standardized tools: EuroQol 5-Dimensions Youth version (EQ-5D-Y).

The EQ-5D-Y test is a tool used to assess health-related quality of life in children and adolescents [33]. It is an adapted version of the EQ-5D test specifically designed for this age group (4–16 years old, but it also can be used in younger children). The test evaluates health across five primary dimensions: 1. mobility; 2. self-care; 3. usual activities; 4. pain/discomfort; and 5. anxiety/depression. Each dimension has three severity levels: none, some, and severe. These responses can be converted into a numeric index where 1 represents the best possible health state, and 3 represents a severe impairment.

The EQ-5D-Y test results provide an overview of the overall health and well-being of the assessed child or adolescent. The score obtained from the EQ-5D-Y test can vary depending on the individual’s responses in each of the five dimensions assessed. The Visual Analog Scale (VAS) is a scale used to measure an individual’s perceived well-being subjectively. In the context of the EQ-5D-Y, the VAS assesses the individual’s self-rated overall health. The individual was asked to mark on a straight line from 0 to 100 how they rate their current health, where 0 represents the worst imaginable health state, and 100 represents the best imaginable health state. The questionnaire was filled out by a third party (e.g., a parent, caregiver or health professional). The measurements marked on the scale were rounded to the nearest number ending in 0 or 5.

We determined the oral health of the patients in our sample through the OHAT scale [34], a tool used by healthcare professionals to assess the oral health status of individuals. The OHAT evaluates various aspects of oral health through a series of categories. Each category is examined and rated on a scale to determine the current state of the patient’s oral health. The typical categories include the following: Lips: Assessment for lesions, ulcers, or dryness. Tongue: Examination of the surface of the tongue for color changes, lesions, or signs of infection. Gums and Soft Tissues: Checking for inflammation, bleeding, ulceration, or color changes. Saliva: Evaluation of the quantity and quality of saliva, considering signs of xerostomia (dry mouth). Natural Teeth: Examination for the presence of caries, plaque, tartar, missing or damaged teeth. Dentures: Checking the fit of dentures, presence of pressure ulcers, or other denture-related issues. Oral Cleanliness: Assessment of the general cleanliness of the mouth and teeth. Dental Pain: Detection of any signs of pain or discomfort in the mouth.

During the assessment, each category is rated based on observed conditions. The ratings help identify areas that require attention or further investigation by dental professionals. The process is as follows: Preparation: Ensure that all necessary tools and materials are available, including gloves, a flashlight, and a mouth mirror. Observation: Carefully observe each category, taking note of any abnormalities or issues. Rating: Assign a rating to each category based on predefined criteria (e.g., healthy, changes, unhealthy). Documentation: Record the findings and ratings for each category. Action Plan: Develop a plan for addressing any identified issues, which may involve referrals to a dentist or implementation of specific oral care routines.

The OHAT is crucial for identifying oral health issues early, which can prevent more severe problems, providing a consistent method for regular monitoring of oral health, especially in vulnerable populations, helping caregivers and healthcare providers improve the quality of oral care provided to patients. Overall, the OHAT is a valuable tool for maintaining and improving oral health in settings where individuals may have limited access to regular dental care. The oral status assessment will always be carried out by the same dental specialist in a sensory-adapted dental room expressly set up for patients with ASD. The determination of oral health will be carried out through the Italian adaptation. The clinical dental examination was performed by Dr. Antonio Fallea, a dentist with specialized training. The assessment of oral health was conducted by Dr. Fallea in a specially equipped dental room designed for patients with ASD. Dental assistants supported Dr. Fallea in managing young patients and ensured that the sensory-adapted dental room was properly organized and utilized.

Questionnaires were administered in written form. Informed consent and assent were obtained from parents/guardians and participants. Parents/guardians completed the consent. The study was conducted in accordance with the Declaration of Helsinki and approved by the local ethics committee “Comitato Etico IRCCS Sicilia-Oasi Maria SS” on 15 March 2023, approval code: 2023/15/03/CE-IRCCS-OASI/253.

### 2.3. Statistical Analyses

Statistical analysis was conducted using RStudio version 2023.03.1 and the R programming language to explore the relationship between oral health status and quality of life in children and adolescents with autism spectrum disorder (ASD). The study dataset included 163 participants, with data on Oral Health Assessment Tool (OHAT) scores, age, sex, autonomy levels, EQ-5D-Y total scores, and Visual Analog Scale (VAS) scores. This information was also incorporated into the analyses for a subset of 90 participants with available IQ data.

Initially, a simple linear regression was applied to evaluate the correlation between OHAT total scores and various explanatory variables, including quality-of-life tests, levels of autonomy, and IQ. A multiple linear regression followed this to ascertain the unique contributions of these variables while adjusting for age and sex. 

Furthermore, to assess the effects of different oral health statuses, participants were stratified into three categories based on their OHAT total scores: Healthy (0–3), Changes and Monitoring (4–8), and Unhealthy (9–16). We tested the normality of the OHAT scores using the D’Agostino-Pearson test [35]. Since the data met the assumptions for ANOVA, we used this test to compare EQ-5D-Y total scores among the groups (EQ-5D-Y responses are reported in Table S1 in Supplementary Material). Tukey’s post hoc comparisons were performed to identify specific differences between groups while controlling for multiple comparisons. EQ-5D-Y responses are detailed in Table S1 in the Supplementary Material.

## 3. Results

### 3.1. Linear Regression

The linear regression analysis revealed that oral health, measured via the OHAT Oral scale, had a statistically significant positive correlation with quality-of-life scores (EQ-5D-Y) in children and adolescents with ASD, with a *p*-value of 0.0027. This model alone accounted for 4.89% of the score variance.

When age and sex were included as covariates, the model’s predictive validity improved. Age explained 9.73% of the variance in quality-of-life scores, with the inclusion of age marginally significant (*p* = 0.0551) and sex non-significant (*p* = 0.1153). The analysis also revealed a statistically significant negative relationship between the OHAT total scores and VAS scores, indicating that better oral health corresponds to lower VAS scores (*p* = 0.0336).

A multiple linear regression was conducted to assess the impact of individual OHAT subscales on the overall quality of life as measured by the EQ-5D-Y total score. The model included the following OHAT subscales as predictors: Lips, Tongue, Gums and Tissues, Saliva, Natural Teeth, Dentures, Oral Cleanliness, and Dental Pain (see Table 2). The model was statistically significant (F(8,153) = 2.841, *p* = 0.0057), with an adjusted R^2^ = 0.0838. Significant predictors included Oral Cleanliness (β = 0.688, *p* = 0.0167) and Saliva (β = −0.561, *p* = 0.0483). Oral Cleanliness was positively associated with EQ-5D-Y scores, while Saliva was inversely associated. Other subscales did not reach statistical significance. Descriptive statistics for EQ-5D-Y scores and VAS are reported in Table 3.

The multiple linear regression analysis assessing the relationship between OHAT subscales and VAS scores also revealed statistical significance (F(8,153) = 2.285, *p* = 0.0244), with an adjusted R^2^ = 0.060. Notably, Dentures (β = −12.21, *p* = 0.0377) and Dental Pain (β = −6.5, *p* = 0.0191) were both negatively associated with VAS.

In a focused analysis of 90 participants, the linear regression analysis showed that IQ was not significantly associated with OHAT total scores (β = 0.02241, *p* = 0.191), explaining a tiny proportion of variance in oral health outcomes (adjusted R^2^ = 0.00818).

Adjusting for age and sex, the analysis found that age was significantly associated with OHAT total scores (β = 0.41403, *p* < 0.0001). Table 4 presents a synthesized view of the relationships between the OHAT total scores and various predictors within the study population.

### 3.2. ANOVA

To determine the impact of oral health status on the quality of life in children and adolescents with autism spectrum disorder (ASD), participants were categorized into three groups based on their OHAT scores: Healthy (0–3), Changes and Monitoring (4–8), and Unhealthy (9–16).

Initially, we tested the normality of the OHAT data using the D’Agostino-Pearson test [35] confirming that the data were normally distributed. Consequently, ANOVA was appropriately applied, revealing a significant overall difference among the groups (*p* = 0.00335).

Following the significant ANOVA result, Tukey post hoc comparisons were conducted to identify specific group differences. The Tukey post hoc analysis revealed significant differences in EQ-5D-Y total scores between the Unhealthy and Healthy groups (*p* = 0.00246). On average, participants with an Unhealthy oral health status reported EQ-5D-Y total scores that were 1.43 points higher compared to those with a Healthy oral health status. This result, summarized in Table 5, highlights the considerable impact of poor oral health on the overall quality of life in individuals with ASD.

There was also a trend towards a significant difference between the Changes and Monitoring group and the Healthy group, suggesting a potential influence of oral health changes on quality of life. However, this did not reach conventional statistical significance. No statistically significant difference was observed between the Unhealthy and Changes and Monitoring groups (*p* = 0.1507).

This table presents the results of Tukey’s post hoc analysis following an ANOVA, comparing the EQ-5D-Y total scores among different OHAT groups (“Healthy”, “Changes and Monitoring”, and “Unhealthy”). The table includes the mean differences (diff), the lower and upper bounds of the 95% confidence intervals (lwr and upr), and the adjusted *p*-values (p adj) for each comparison.

## 4. Discussion

An initial exploration of the relationship between oral health and quality of life was undertaken using a simple linear regression model. The model assessed the impact of the OHAT Oral scale on EQ-5D-Y total scores, revealing a statistically significant positive association (β = 0.13045, *p* = 0.00271). This suggests that better oral health is associated with higher quality-of-life scores and confirms the results of previous studies [3,26]. Furthermore, the results obtained, despite the lack of a control sample, demonstrate that they are in line with previous studies. For example, a 2011 study revealed that children with autism (*n* = 61) showed a higher prevalence of tooth decay, poor oral hygiene, and extensive unmet need for dental treatment compared to the healthy non-autistic control group (*n* = 61) [35]. There is no existing literature that uses the OHAT scale specifically for people with ASD. Only Ahmed M. Bokhari et al. administered the OHAT scale to a group of 478 adolescents and found the following: 80.3% of school children had poor tooth conditions, and 36.2% of school children frequently suffered from toothache [36]. Furthermore, our results also confirm the importance of oral health for the overall well-being of individuals with autism.

A multiple linear regression model was fitted to elucidate the relationship further while accounting for potential confounding factors. This adjusted model included age and sex as covariates. The results indicated that the association between oral health and quality of life persisted after adjustment, with a slightly attenuated effect size (β = 0.10536, *p* = 0.0167). Additionally, age demonstrated a marginally significant positive association with quality-of-life scores. This result does not confirm what was highlighted in other studies in which children in the older age group were shown to have a poorer quality of life, and their impact on the family was also more significant [3].

The positive association between Oral Cleanliness and EQ-5D-Y total scores suggests that maintaining oral hygiene and dental cleanliness plays a crucial role in enhancing overall well-being in children with ASD. Conversely, the negative association between Saliva and EQ-5D-Y scores may reflect the challenges in managing the abundant salivary flow, typically described as sialorrhea, which is common in patients with autism spectrum disorder and could negatively impact comfort and overall oral hygiene quality.

The significant negative impact of Dentures and Dental Pain on VAS scores highlights the importance of addressing specific oral health issues to improve subjective health perceptions. The presence of dentures (which in this study was 0 for all patients, although they may be found in patients with syndromic dysontogenesis comorbid with autism spectrum disorders) [37,38] and experiences of dental pain appear to be critical factors that adversely affect health perceptions in children with ASD, further emphasizing the need for targeted oral health interventions.

Regarding the EQ-5D-Y scale, specific data on autistic patients are not available. However, when applied to ID, it has shown the following properties: a caregiver-completed proxy version of the EQ-5D-Y-5L, when applied to children with ID, has good distributional properties with extremely few (<1%) missing values and without ceiling effects. Compared with other populations, children with ID score poorly on self-care, usual activities, and worry/sadness/unhappiness. Convergent validity is good for most dimensions and for the EQ-VAS. Test-retest reliability is fair to substantial for four dimensions but poor for pain/discomfort and EQ-VAS, indicating variable health states in some children. Although ID is not a disease state, these children’s EQ-5D-Y-5L scores indicate severe problems affecting health-related quality of life compared with most other populations, particularly in regard to self-care, usual activities, and worry/sadness/unhappiness dimensions [39].

Our findings suggest that the “Autonomy” subscale of the EQ-5D-Y has a positive relationship with the “OHAT Oral Scale”. It has been described that children with autism are not autonomous in carrying out oral hygiene practices and often need help from caregivers. This dependence represents a challenging aspect of the daily routine. It is not prioritized by caregivers of children with ASD, who prioritize other essential tasks such as nutrition and meeting different needs. Studies report that children’s heavy dependence raises parental burnout levels. In addition, given that individuals with ASD are often unable to perform their oral hygiene, they may not tolerate the act of brushing their teeth; parents may have to resort to restrictions for their children, which can be physically and emotionally demanding [40,41]. Dependence on caregivers and lack of autonomy in personal self-care habits are barriers to oral health [42]. As children age, they have more independence in carrying out routine daily activities, such as oral hygiene [43].

Parents of autistic children showed far more difficulty in brushing their children’s teeth and more significant physical effort in helping them compared to parents of non-autistic children. This is due to the lack of manual dexterity of people with ASD [42] and insufficient knowledge about oral health [44].

The percentage of children who required assistance from an adult to brush their teeth and did not do so independently ranged from 64.2% [13] to 68.0% [43].

Our results regarding the correlation between IQ and oral health are in line with some more recent studies. In fact, these show that in this specific ASD population, the IQ variable does not significantly influence dental health status [45].

A study that aimed to determine the state of oral health among different abled individuals divided into groups according to IQ found that these subjects had a fair amount of oral hygiene, and it was not statistically related to IQ levels. This could be attributed to the help they were receiving [46]. In addition, another study explicitly conducted on autistic children showed that they had caries rates similar to those of their functionally independent peers [47]. By evaluating the prevalence of gingivitis or dental caries as indicators, it has been shown that there is a statistically significant relationship between IQ and the prevalence of moderate gingivitis and no significant relationship between IQ and dental caries [48].

It should be noted that our study concerned a specific ASD population. In contrast, according to other studies in which only IQ was considered to show that the presence of caries is more significant in the lower IQ group, people with a lower IQ were more likely to have worse oral health [49]. Another study showed that the level of tooth decay was significantly higher in older subjects than in those of younger age and in subjects with severe ID than in those with mild ID [50]. Still, others have shown that there was a statistically significant lower level of tooth decay and dental plaque in the higher-level intelligence group [51].

Looking at other studies that have evaluated this correlation in general, we found that more complex dental problems are more frequently described than oral health in general. IQ level could help manage dental anxiety (DA) and maintain an excellent oral health-related quality of life in children [43]. In a study conducted in India on 202 children, which precisely assessed the quality of life related to oral health (OHRQoL), dental anxiety (DA) and IQ, the results showed that DA was negatively correlated with IQ (r = −0.093) and OHRQoL (r = −0.065), but was not statistically significant. DA was negatively correlated with IQ and OHRQoL. In line with our results, the gender-based comparison revealed no significant differences in the distribution of girls and boys within the different grades of IQ (*p* = 0.74), DA (*p* = 0.29) and OHRQoL (*p* = 0.85) levels [52].

## 5. Conclusions

The findings emphasize the significance of oral health maintenance, monitoring, and potential interventions to enhance the overall well-being of children and adolescents with ASD. Unhealthy oral health conditions significantly impact the overall quality of life in individuals with ASD. Therefore, it will be of great importance to implement periodic dental assessments and subsequent dental treatments for patients with autism within the therapeutic protocols. While not directly assessed in this study, treatments should include appropriate desensitization of dental anxiety and sensory-adapted procedures, based on established best practices to effectively address the unique needs of children with autism.

## Figures and Tables

**Table 1:** Criteria for diagnosis of autism spectrum disorder from the DSM-V.

	Criteria
A	Persistent deficits in social communication and social interaction across multiple contexts
B	Restricted, repetitive patterns of behavior, interests, or activities
C	Symptoms must be present in early childhood (but may not become fully manifest until social demands exceed limited capacities)
D	Symptoms must be present in the early developmental period (but may not become fully manifest until social demands exceed limited capacities or may be masked by learned strategies in later life)
E	Symptoms cause clinically significant impairment in social, occupational, or other important areas of current functioning.
F	These disturbances are not better explained by intellectual disability (intellectual developmental disorder) or global developmental delay. Intellectual disability and autism spectrum disorder frequently co-occur; to make comorbid diagnoses of autism spectrum disorder and intellectual disability, social communication should be below that expected for general developmental level.

**Table 2:** Descriptive statistics for OHAT categories. The table reports the mean ± standard error (SE) and the range for each category. The range provides a measure of the dispersion of the data, while the SE indicates the precision of the mean estimates for study participants (age 7.83 ± 0.44).

Category	Mean ± SE	Range
Lips	1.46 ± 0.05	0–2
Tongue	1.27 ± 0.05	0–2
Gums and Tissues	1.34 ± 0.05	0–2
Saliva	1.10 ± 0.06	0–2
Natural Teeth	0.45 ± 0.06	0–2
Dentures	0.02 ± 0.01	0–2
Oral Cleanliness	1.33 ± 0.06	0–2
Dental Pain	0.12 ± 0.03	0–2
Total OHAT	7.08 ± 0.26	1–14

**Table 3:** Descriptive statistics for EQ-5D-Y scores and VAS including mean values with standard errors (SE) and ranges.

Category	Mean ± SE	Range
Mobility	1.15 ± 0.03	1–3
Looking After Myself	2.31 ± 0.05	1–3
Usual Activities	1.76 ± 0.06	1–3
Pain or Discomfort	1.24 ± 0.04	1–3
Mood	1.43 ± 0.05	1–3
EQ-5D-Y Total	7.81 ± 0.15	1–14
VAS	83.57 ± 1.02	30–100

**Table 4:** The regression coefficients indicate the predicted change in OHAT total scores for a one-point increase in the relevant explanatory variable.

Model	Explanatory Variable	F	df	*p* Value	R² Adjusted
1	EQ-5D-Y	9.28	1, 160	<0.001	4.893%
2	EQ-5D-Y, Age, sex	5.54	3, 158	<0.001	9.73%
3	Autonomy	3.081	1, 160	>0.05	1.3%
4	VAS	4.593	1, 160	<0.05	2.2%
5	IQ	1.73	1, 88	>0.05	0.82%
6	IQ, Age, Sex	5.927	4, 85	<0.0001	18.13%

**Table 5:** Tukey multiple comparisons of means for EQ-5D-Y total scores across OHAT groups.

Comparison	Mean ± SE	Range	Difference (diff)	Lower Bound (lwr)	Upper Bound (upr)	Adjusted *p*-Value (p adj)
Changes and Monitoring–Healthy	2.25 ± 0.218	1–3	0.852	−0.113	1.817	0.095
Unhealthy–Healthy	5.72 ± 0.211	4–8	1.432	0.435	2.429	0.002
Unhealthy–Changes and Monitoring	10.6 ± 0.218	9–14	0.580	−0.154	1.313	0.151

## Data Availability

The data presented in this study are available on request from the corresponding author.

## Supplementary Materials

The following supporting information can be downloaded at: https://www.mdpi.com/article/10.3390/jcm13175179/s1, Table S1: EQ-5D-Y responses, Table S2: Oral Health Assessment Tool scores.
