# Supplementary material for: Oral Health and Quality of Life in People with Autism Spectrum Disorder
Source: J Clin Med. 2024 Aug 31;13(17):5179. doi: 10.3390/jcm13175179 (PMC11396441; doi:10.3390/jcm13175179)
Supplement: Supplementary file 1 [file jcm-13-05179-s001.zip › jcm-3088718-supplementary.pdf]

Table S1. EQ-5D-Y responses

N.	Mobility	Looking after myself	Usual activities	Pain or discomfort	Worried, sad or unhappy	EQ-5D-Y Total	visual analogue scale
**1**	1	2	1	1	1	6	80
**2**	2	2	1	1	2	8	80
**3**	1	2	3	2	3	11	50
**4**	2	3	2	1	1	10	85
**5**	2	3	2	2	2	11	60
**6**	2	2	1	1	2	8	90
**7**	1	2	1	1	2	7	80
**8**	1	2	2	1	1	7	95
**9**	1	3	2	2	2	10	70
**10**	2	3	3	1	2	11	50
**11**	1	3	3	2	1	10	50
**12**	1	3	3	1	2	10	75
**13**	1	3	1	1	1	7	90
**14**	1	2	2	2	1	8	90
**15**	1	2	2	1	1	7	90
**16**	1	2	2	2	2	9	80
**17**	1	3	3	1	1	9	95
**18**	1	2	2	1	2	8	60
**19**	1	3	2	1	1	8	85
**20**	1	3	2	1	1	8	80
**21**	2	3	2	1	1	9	80
**22**	1	2	1	1	1	6	90
**23**	1	3	3	1	1	9	100
**24**	1	2	1	1	1	6	90
**25**	1	3	3	1	1	9	100
**26**	1	3	3	2	1	10	90
**27**	2	3	2	1	2	10	65
**28**	1	2	1	2	1	7	90
**29**	1	2	2	1	1	7	90
**30**	0	0	0	0	0	0	0
**31**	1	1	1	1	1	5	100
**32**	1	1	1	1	3	7	95
**33**	1	1	1	1	1	5	100
**34**	1	3	2	2	2	10	60
**35**	2	3	3	1	2	11	70
**36**	1	3	2	1	1	8	85
**37**	1	2	2	1	1	7	90
**38**	1	2	2	1	1	7	80
**39**	1	2	1	1	1	6	100
**40**	1	3	1	1	1	7	100

**41**	1	2	2	1	1	7	90
**42**	1	3	2	1	2	9	80
**43**	1	3	2	1	1	8	50
**44**	1	2	2	1	1	7	90
**45**	2	3	2	1	2	10	90
**46**	1	3	2	2	2	10	80
**47**	1	2	1	1	2	7	70
**48**	1	3	1	2	2	9	70
**49**	1	1	1	1	2	6	80
**50**	2	2	2	1	1	9	80
**51**	1	1	1	1	1	5	100
**52**	2	1	1	1	1	6	90
**53**	1	3	3	1	3	11	90
**54**	1	3	3	1	3	11	90
**55**	1	1	1	2	2	7	70
**56**	1	3	2	1	2	9	50
**57**	1	2	2	2	2	9	80
**58**	1	2	2	1	2	8	70
**59**	1	3	1	1	1	7	95
**60**	1	3	2	3	1	10	55
**61**	2	3	2	1	1	9	70
**62**	1	3	2	2	1	9	85
**63**	1	2	2	1	1	7	85
**64**	1	2	2	1	1	9	80
**65**	1	2	2	1	1	7	90
**66**	1	3	3	1	1	9	80
**67**	1	3	3	3	3	13	90
**68**	1	3	2	1	1	8	80
**69**	2	1	1	1	1	6	90
**70**	1	1	1	1	1	5	95
**71**	1	3	1	2	1	8	100
**72**	1	3	1	1	1	7	85
**73**	1	3	1	1	1	7	90
**74**	1	3	2	2	2	10	90
**75**	1	1	1	1	1	5	90
**76**	1	2	2	1	2	8	80
**77**	1	3	2	1	1	8	85
**78**	1	1	1	1	1	5	95
**79**	1	3	3	2	2	11	60
**80**	1	2	2	1	2	8	90
**81**	1	2	2	1	2	8	95
**82**	1	1	1	1	1	5	100
**83**	1	2	1	1	1	6	70

**84**	1	2	2	1	1	7	80
**85**	1	3	1	1	1	7	90
**86**	1	2	2	1	2	8	85
**87**	1	2	1	1	2	7	90
**88**	1	2	2	1	1	7	90
**89**	1	2	1	1	1	6	90
**90**	1	3	2	2	1	9	85
**91**	1	2	2	1	1	7	85
**92**	2	2	1	2	2	9	90
**93**	1	3	2	2	2	10	90
**94**	1	2	1	1	1	6	90
**95**	1	2	2	1	1	7	90
**96**	1	2	2	1	1	7	90
**97**	1	2	2	1	2	8	90
**98**	2	2	2	1	1	8	85
**99**	2	3	3	1	2	11	70
**100**	1	2	1	1	1	6	80
**101**	1	2	2	1	1	7	80
**102**	1	2	1	1	1	1	80
**103**	1	3	2	2	1	9	80
**104**	1	1	1	1	3	7	90
**105**	1	3	2	1	1	8	90
**106**	1	2	3	2	3	11	70
**107**	1	2	2	1	1	7	95
**108**	1	2	2	1	1	7	90
**109**	1	3	1	1	1	7	85
**110**	1	2	1	2	1	7	90
**111**	1	3	3	3	2	1	90
**112**	1	3	2	1	1	8	90
**113**	1	2	1	2	2	8	80
**114**	1	2	1	1	1	6	100
**115**	1	1	2	1	1	6	95
**116**	2	3	3	1	1	10	80
**117**	1	2	2	2	1	8	60
**118**	1	1	1	1	1	5	95
**119**	1	2	2	1	2	8	85
**120**	1	2	2	2	1	8	75
**121**	1	3	2	1	2	9	90
**122**	1	1	1	1	1	5	95
**123**	2	3	2	1	2	10	80
**124**	2	3	2	1	2	10	70
**125**	1	3	2	1	2	9	95
**126**	2	2	1	1	2	8	90

**127**	1	2	1	1	2	7	90
**128**	1	3	3	1	2	10	95
**129**	1	3	2	1	1	8	70
**130**	1	2	1	1	1	6	95
**131**	1	3	2	1	1	8	100
**132**	1	2	1	1	1	6	95
**133**	1	1	1	1	1	5	80
**134**	2	3	2	1	2	10	90
**135**	1	2	1	1	1	6	50
**136**	1	3	1	1	1	7	90
**137**	1	1	2	1	3	8	60
**138**	1	2	2	1	2	8	90
**139**	1	3	3	1	1	9	100
**140**	1	3	1	1	1	7	90
**141**	1	3	1	1	1	7	90
**142**	1	2	1	2	3	9	80
**143**	1	2	1	1	1	6	95
**144**	1	2	2	2	1	8	90
**145**	1	2	1	2	1	7	70
**146**	3	3	3	2	3	14	30
**147**	1	3	1	1	1	7	90
**148**	1	2	2	1	2	8	80
**149**	1	1	1	1	1	5	90
**150**	1	3	3	1	1	9	95
**151**	1	2	1	2	1	7	90
**152**	1	3	2	1	1	8	80
**153**	1	2	2	1	1	7	90
**154**	2	3	1	1	2	9	50
**155**	1	3	3	2	1	10	60
**156**	1	2	3	1	2	9	99
**157**	1	2	1	1	1	6	95
**158**	1	2	2	1	1	7	95
**159**	1	2	1	1	1	6	90
**160**	1	3	3	1	1	9	70
**161**	1	1	1	2	2	7	100
**162**	1	3	1	1	1	7	90
**163**	1	2	1	1	2	7	80

Table S2. Oral Health Assessment Tool scores

N.	Total Score	Lips	Tongue	Gums and tissues	Saliva	Natural teeth Yes/No	Dentures Yes/No	Oral cleanliness	Dental pain
**1**	4	1	1	1	1	0	0	0	0
**2**	2	0	0	1	0	0	0	1	0
**3**	3	1	0	1	0	0	0	0	1
**4**	8	2	2	2	1	0	0	1	0
**5**	12	2	2	2	2	2	0	2	0
**6**	5	1	1	1	1	0	0	1	0
**7**	6	1	1	1	1	1	0	1	0
**8**	9	2	1	2	1	1	0	2	0
**9**	9	2	2	1	1	1	0	2	0
**10**	6	1	1	1	1	0	0	2	0
**11**	8	2	1	1	1	2	0	1	0
**12**	11	2	2	2	2	1	0	2	0
**13**	4	1	1	1	0	0	0	1	0
**14**	5	1	1	1	1	0	0	1	0
**15**	4	1	0	1	0	0	0	2	0
**16**	8	2	2	2	1	0	0	1	0
**17**	6	2	1	1	1	0	0	1	0
**18**	10	2	2	2	1	1	0	2	0
**19**	9	2	1	2	2	0	0	2	0
**20**	6	1	1	1	2	0	0	1	0
**21**	1	0	0	1	0	0	0	0	0
**22**	12	2	2	2	2	1	0	2	0
**23**	8	2	2	2	1	0	0	1	0
**24**	9	2	2	2	1	0	0	2	0
**25**	8	2	1	2	1	1	0	1	0
**26**	11	2	2	2	2	1	0	2	0
**27**	4	0	1	1	1	0	0	1	0
**28**	4	1	1	1	1	0	0	0	0
**29**	9	2	2	2	1	0	0	2	0
**30**	0	0	0	0	0	0	0	0	0
**31**	4	1	1	1	0	0	0	1	0
**32**	4	1	1	1	0	0	0	1	0
**33**	9	2	2	1	2	0	0	2	0
**34**	6	1	1	1	1	0	0	2	0
**35**	6	1	1	1	1	0	0	2	0
**36**	1	0	0	0	1	0	0	0	0
**37**	12	2	2	2	2	2	0	2	0
**38**	5	1	2	1	0	0	0	1	0
**39**	10	2	2	2	2	0	0	2	0
**40**	8	2	2	1	1	0	0	2	0

**41**	2	1	0	1	0	0	0	0	0
**42**	11	2	2	2	1	2	0	2	0
**43**	2	1	0	1	0	0	0	0	0
**44**	5	1	1	1	1	0	0	1	0
**45**	11	2	2	2	2	1	0	2	0
**46**	13	2	2	1	2	2	0	2	2
**47**	5	1	1	1	1	0	0	1	0
**48**	7	2	1	2	1	0	0	1	0
**49**	10	2	1	1	1	2	0	2	1
**50**	5	2	1	1	0	0	0	1	0
**51**	5	1	1	1	1	0	0	1	0
**52**	9	2	2	1	2	0	0	2	0
**53**	5	1	1	1	0	0	0	2	0
**54**	6	2	2	1	0	0	0	1	0
**55**	4	1	1	1	0	0	0	1	0
**56**	4	1	1	1	0	0	0	1	0
**57**	6	2	1	1	1	0	0	1	0
**58**	5	1	1	1	1	0	0	1	0
**59**	8	1	2	2	1	0	0	2	0
**60**	10	2	2	2	2	0	0	2	0
**61**	10	2	2	1	1	2	0	2	0
**62**	14	2	2	2	2	2	0	2	2
**63**	10	2	2	2	2	0	0	2	0
**64**	11	2	2	2	1	2	0	2	0
**65**	2	1	1	0	0	0	0	0	0
**66**	9	2	1	1	1	2	0	2	0
**67**	11	2	2	2	2	0	0	2	1
**68**	8	2	1	2	1	0	0	2	0
**69**	3	0	1	1	0	0	0	1	0
**70**	8	2	2	2	1	0	0	1	0
**71**	2	1	0	0	0	0	0	1	0
**72**	6	2	1	1	1	0	0	1	0
**73**	6	1	1	2	0	1	0	1	0
**74**	7	2	1	2	1	0	0	1	0
**75**	1	1	0	0	0	0	0	0	0
**76**	11	2	2	1	2	2	0	2	0
**77**	4	1	1	1	1	0	0	0	0
**78**	13	2	2	2	2	2	0	2	1
**79**	2	1	0	1	0	0	0	0	0
**80**	5	1	1	1	1	0	0	1	0
**81**	10	2	2	2	2	0	0	2	0
**82**	9	2	2	1	1	1	1	1	0
**83**	10	1	1	2	2	2	0	2	0

**84**	8	1	1	2	1	2	0	1	0
**85**	1	0	0	0	1	0	0	0	0
**86**	4	1	0	1	0	0	0	2	0
**87**	11	2	2	2	2	1	0	2	0
**88**	10	2	2	2	2	0	0	2	0
**89**	10	2	2	2	1	1	0	2	0
**90**	2	1	0	1	0	0	0	0	0
**91**	13	2	2	2	2	2	0	2	1
**92**	3	1	0	1	0	0	0	1	0
**93**	11	2	2	1	2	2	0	2	0
**94**	13	2	2	2	2	2	0	2	1
**95**	2	1	1	0	0	0	0	0	0
**96**	7	2	2	1	1	0	0	1	0
**97**	11	2	2	2	2	0	0	2	1
**98**	7	2	1	2	1	0	0	1	0
**99**	12	2	2	2	2	2	0	2	0
**100**	12	2	2	2	2	2	0	2	0
**101**	8	1	1	1	1	0	0	2	2
**102**	7	2	2	1	1	0	0	1	0
**103**	2	1	1	0	0	0	0	0	0
**104**	5	1	1	1	1	0	0	1	0
**105**	6	2	1	1	1	0	0	1	0
**106**	10	2	2	2	2	0	0	2	0
**107**	4	0	0	0	0	2	0	2	0
**108**	2	1	0	0	1	0	0	0	0
**109**	12	2	2	2	2	2	0	2	0
**110**	7	1	1	2	1	0	0	2	0
**111**	4	2	1	0	0	0	0	1	0
**112**	12	2	2	2	2	2	0	2	0
**113**	10	2	2	2	2	1	0	1	0
**114**	3	0	0	1	1	0	0	1	0
**115**	10	2	2	2	2	0	0	2	0
**116**	10	2	2	2	2	0	0	2	0
**117**	8	1	1	2	1	1	0	1	1
**118**	4	0	1	1	0	0	0	1	1
**119**	4	1	1	1	1	0	0	0	0
**120**	5	1	1	1	1	0	0	1	0
**121**	9	2	2	1	2	0	0	2	0
**122**	7	1	2	2	1	0	0	1	0
**123**	12	2	2	2	2	2	0	2	0
**124**	11	2	1	2	2	2	0	2	0
**125**	10	2	2	2	2	0	0	2	0
**126**	3	1	0	0	1	0	0	1	0

**127**	9	2	2	1	2	0	0	2	0
**128**	6	1	1	1	1	0	0	1	1
**129**	8	2	1	2	1	0	0	2	0
**130**	6	1	1	1	1	0	0	2	0
**131**	8	2	2	1	1	0	0	2	0
**132**	1	0	0	0	1	0	0	0	0
**133**	7	1	0	1	2	0	0	2	1
**134**	6	2	1	1	1	0	0	1	0
**135**	6	1	1	1	0	2	0	1	0
**136**	8	1	1	2	2	0	0	2	0
**137**	6	2	1	1	1	0	0	1	0
**138**	2	1	1	0	0	0	0	0	0
**139**	10	2	2	2	1	0	0	2	1
**140**	6	1	1	2	1	0	0	1	0
**141**	11	2	1	2	2	1	2	1	0
**142**	3	1	1	1	0	0	0	0	0
**143**	3	1	1	1	0	0	0	0	0
**144**	10	2	2	2	2	0	0	2	0
**145**	10	2	2	2	2	0	0	2	0
**146**	7	1	1	2	2	0	0	1	0
**147**	6	1	1	1	1	0	0	2	0
**148**	8	1	1	2	1	1	0	2	0
**149**	5	1	1	1	1	0	0	1	0
**150**	12	2	2	2	2	2	0	2	0
**151**	5	1	1	1	1	0	0	1	0
**152**	10	2	2	2	2	0	0	2	0
**153**	10	2	2	2	2	0	0	2	0
**154**	1	0	0	1	0	0	0	0	0
**155**	3	1	0	1	1	0	0	0	0
**156**	3	1	1	1	0	0	0	0	0
**157**	8	2	1	2	1	0	0	2	0
**158**	6	2	1	1	1	0	0	1	0
**159**	5	1	1	1	1	0	0	1	0
**160**	4	1	1	0	1	0	0	1	0
**161**	12	2	2	2	2	2	0	2	0
**162**	12	2	2	2	2	2	0	2	0
**163**	11	2	2	2	2	0	0	2	1
